# On‐Chip Micro‐Pseudocapacitors for Ultrahigh Energy and Power Delivery

**DOI:** 10.1002/advs.201500067

**Published:** 2015-04-02

**Authors:** Jiuhui Han, Yu‐Ching Lin, Luyang Chen, Yao‐Chuan Tsai, Yoshikazu Ito, Xianwei Guo, Akihiko Hirata, Takeshi Fujita, Masayoshi Esashi, Thomas Gessner, Mingwei Chen

**Affiliations:** ^1^ WPI Advanced Institute for Materials ResearchTohoku UniversitySendai980–8577Japan; ^2^ MEMSCORE CooperationSendai981–3206Japan; ^3^ Micro System Integration CenterTohoku UniversitySendai980–0845Japan; ^4^ Fraunhofer Institute for Electronic Nano SystemsChemnitz09126Germany; ^5^ State Key Laboratory of Metal Matrix CompositesSchool of Materials Science and EngineeringShanghai Jiao Tong UniversityShanghai200030P. R China; ^6^ CRESTJST4–1–8 Honcho KawaguchiSaitama332–0012Japan

**Keywords:** energy storage, manganese oxide, microdevice, nanoporous gold, supercapacitor

## Abstract

**Microscale supercapapcitors** based on hierarchical nanoporous hybrid electrodes consisting of 3D bicontinuous nanoporous gold and pseudocapacitive manganese oxide deliver an excellent stack capacitance of 99.1 F cm^−3^ and a high energy density of 12.7 mW h cm^−3^ with a retained high power density of 46.6 W cm^−3^.

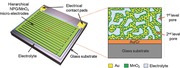

The demands of continuous miniaturization of microelectromechanical systems (MEMS) and portable electronic devices have motivated the efforts on integrating power sources with electronic circuits.^1^ This calls for the electrochemical energy storage devices, such as batteries and supercapacitors, not only to scale down in dimensions to fit on‐chip geometries of integrated circuits but also to be compatible in fabrication methods with current MEMS and semiconductor techniques. In contrast to thin film batteries whose properties drop dramatically with the decrease of sizes, micro‐supercapacitors often have better performance in comparison with their bulk counterparts as the result of reduced transport length of charge and electrolytes.^1^, ^2^ Interdigital planar form micro‐supercapaciotors (MSCs) with active carbon,^3^ carbide‐derived carbon,^4^ carbon nanoparticles/nanotubes^2^, ^5^, ^6^, ^7^ and multilayer graphene^7^, ^8^, ^9^, ^10^, ^11^, ^12^, ^13^ as electrode materials have been developed and are capable of delivering high power density by electrochemical double layer charge storage. However, their energy densities, typically, 0.1–1.0 mW h cm^−3^, are insufficient to meet the requirement of the reasonable operational time of microdevices. Pseudocapacitive materials, such as transition metal oxides (MnO_2_, RuO_2_, etc.) and conductive polymers (polyaniline, polypyrrole, etc.), represent an important branch of electrode materials for supercapacitor applications.^14^ As the charge storage is based on the fast surface redox (faradic) reactions, they have very high theoretical capacitance, for instance, 1370 F g^−1^ for MnO_2_.^15^, ^16^ Indeed, MSCs with pseudocapacitive electrode materials have been reported to have respectable gravimetric and volumetric capacitance.^17^, ^18^, ^19^, ^20^, ^21^, ^22^, ^23^, ^24^, ^25^ Nevertheless, the pseudocapacitive materials usually suffer from low electrical conductivity and, as a result, their achievable capacitances and power performance are far from the theoretical expectation and device requirements. In addition to the demands of an increased conductivity, porous structure design is also important for supercapacitor electrodes because it can provide/allow a large surface area, good accessibility of the ions to the electrochemically active surface and excellent capacitive behaviors even in quick charge‐discharge operations.^26^, ^27^, ^28^, ^29^, ^30^ Despite many efforts made in recent years, it is still the most challenging task to concurrently realize high energy and high power densities in MSCs. Moreover, although many new methods have recently been developed to fabricate MSCs,^6^, ^8^, ^9^, ^13^, ^22^ most of them are incompatible or not fully compatible with current MEMS and semiconductor techniques and, hence, cannot be employed directly to integrate the MSCs into electronic circuits. Here we report a hierarchical nanoporous gold (NPG)/manganese oxide (MnO_2_) hybrid micro‐pseudocapacitor (MPC), fabricated by the standard MEMS technique, with the high power densities comparable to carbon/graphene‐based MSCs and, remarkably, ultrahigh specific energy which is even better than that of commercial thin film Li ion batteries and more than an order of magnitude higher than that of carbon/graphene‐based MSCs.

**Figure** **1**a schematically illustrates the structure of the NPG/MnO_2_ based MPCs. The entire device, with dimensions of 3.95 × 5.45 mm (Table S1, Supporting Information), is settled on a glass substrate and consists of 16 microelectrodes organized in an interdigital planar geometry. Every eight out of the 16 electrodes, made of NPG and MnO_2_, form either the cathode or anode and the gap between two electrodes is 50 μm (Figure 1b,c). The fully integrated on‐chip NPG/MnO_2_ MPCs were fabricated by the standard MEMS technique and the complete fabrication process is schematically illustrated in Figure S1, Supporting Information. The hierarchical NPG was prepared by two‐step dealloying of a electrochemically plated Au_17.7_Ag_82.3_ (at%) precursor (Figure S2, Supporting Information).^31^ In the first step, electrochemical dealloying only partly removed the Ag (≈49% of the entire Ag) and formed a nanoporous AuAg alloy (np‐AuAg) with a nanopore size of ≈10–20 nm. After annealed at 300 °C for 1 h, the coarsened nanoporous alloy was subjected to the second step dealloying to generate small pores in the coarsened AuAg ligaments by further selectively leaching away Ag. As shown in **Figure** **2**a,b and Figure S3, Supporting Information, the hierarchical NPG is uniform in pore distribution and film thickness, and more importantly, crack‐free. The large pores have a size of 200–300 nm and the second‐level small pores are of 20–40 nm. Different from free‐standing AuAg thin films that the volume changes during the removal of Ag can be compensated by the macroscopic shrinkage of the film, the on‐chip AuAg alloy films are horizontally constrained by the solid substrates and hence cracks are easy to form during dealloying,^32^, ^33^ especially when the precursors contain a high concentration of Ag. The flaw‐free porous structure benefits from the two‐step dealloying, which gives rise to a small film thickness shrinkage from precursor 840 ± 30 nm to dealloyed 800 ± 30 nm (Figure S4, Supporting Information) and a limited volume reduction of ≈5%, much lower than the ≈30% volume shrinkage in conventionally dealloyed samples.^34^ On the basis of the composition evolution and the volume contraction of the NPG films, a high porosity of ≈79 vol% can be achieved in the on‐chip hierarchical NPG (Figure 2c).

**Figure 1:**
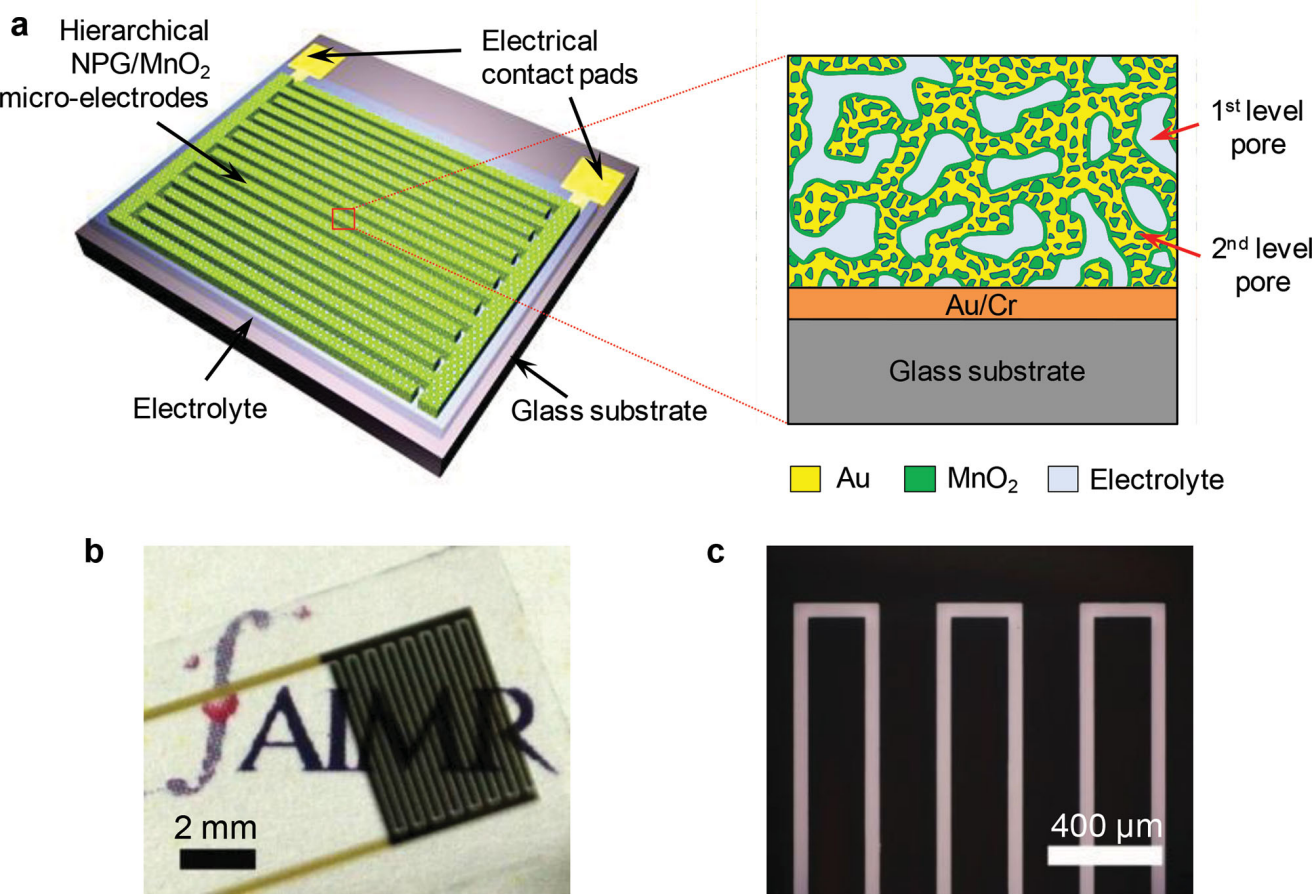
Design of the hierarchical NPG/MnO_2_ MPCs. a) Schematic of the microdevice. Hierarchical NPG/MnO_2_ microelectrodes were grown on planar Au/Cr templates that have been previously fabricated into an interdigital configuration. The final device consists of 16 fingers, every eight out of which were electrically interconnected. NPG with hierarchical porosity provides pore channels for both MnO_2_ plating and electrolyte accommodation. b) A digital photograph of the microdevice. c) Optical image of the interdigital fingers. The two electrodes are separated by a 50 μm channel.

**Figure 2:**
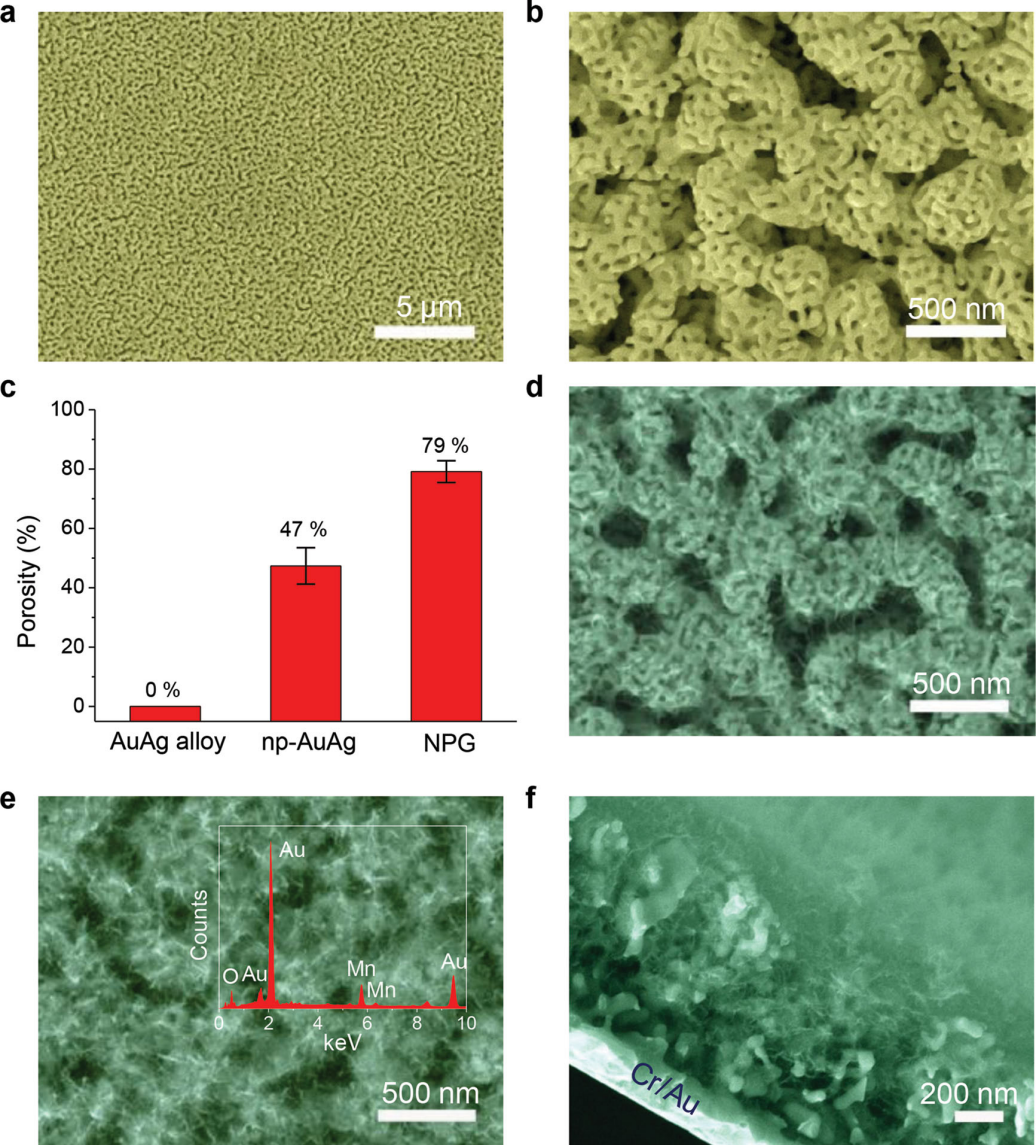
Characterization of the hierarchical NPG/MnO_2_ microelectrodes. a,b) Low and high magnification SEM images for hierarchical NPG, showing the uniform and crack‐free NPG films with bicontinuous porosity on distinctly two different length scales. c) Evolution of porosity during the preparation process. d) SEM image of the hierarchical NPG/MnO_2_ composites with a MnO_2_ plating time of 11 min. Flocculent MnO_2_ were uniformly plated into the nano‐pore channels, leaving open porosity. e,f) Plane‐view and cross‐sectional SEM images of the 15 min‐plated hierarchical NPG/MnO_2_ composite, showing the conformal deposition of MnO_2_ into the deep pore channels of hierarchical NPG. A ≈200 nm thick MnO_2_ film was also formed on the NPG top surface. The inset in (e) shows the EDS spectrum of the NPG/MnO_2_ composite.

Figure 2d,e show the microstructure of the hierarchical NPG electrodes after MnO_2_ deposition (see also Figure S5, Supporting Information). Chemical composition of MnO_2_ is verified by X‐ray energy‐dispersive spectroscopy (EDS) (Figure 2e, inset) and has been demonstrated by detailed XPS and TEM characterizations in our previous studies.^16^, ^35^ It is clear that the flocculent MnO_2_ has been successfully plated into the nano‐pore channels of NPG. For the sample with MnO_2_ plating time up to 11 min (corresponding to an areal density of 74.5 ± 2.1 μg cm^−2^), no accumulation of MnO_2_ can be seen from the top surface of NPG films. This is in obvious contrast to the MnO_2_ growth on regular monolithic NPG. With the same MnO_2_ plating amount, a very thick MnO_2_ film (520 ± 30 nm, Figure S6, Supporting Information) usually forms on the NPG top surface. The exceptional plating performance of hierarchical NPG is due to the large porosity as well as enhanced mass transport by the novel hierarchical porous structure. Cross‐sectional SEM image of the NPG/MnO_2_ composite reveals a conformal electrodeposition of MnO_2_ across the entire 800 nm porous film (Figure 2f), which has also been confirmed by EDS mapping and line sweep (Figures S7,S8, Supporting Information). It is worth noting that after the deposition of MnO_2_, the hybrid film remains the open porosity, even for the 15 min plated NPG/MnO_2_ (MnO_2_ areal density 95.4 ± 4.2 μg cm^−2^) in which a ≈200 nm MnO_2_ film was formed on the NPG top surface (Figure 2e,f). The open pores in the electrode are filled upon the casting of electrolytes. Finally, the microelectrodes are functionalized with interconnected current collector, active material and electrolyte together with minimized lengths for ionic and electric transport in the 3D nanoporous structure.

Electrochemical performance of the hierarchical NPG/MnO_2_ MPCs was firstly evaluated in a 5.0 m LiCl aqueous solution. Cyclic voltammograms (CVs) of the supercapacitors retain a perfectly symmetrical rectangular shape over 0.7 V potential window at scan rates varying from 5 to 100 mV s^−1^ (**Figure** **3**a and Figure S9, Supporting Information). This double‐layer capacitor‐like CV behavior actually originates from the fast, reversible successive surface redox reactions of MnO_2_ by means of proton incorporation, as well as surface electro‐adsorption of Li^+^ cations according to the reaction^14^, ^36^

$${\mathrm{MnO}}_{2}+xH^{+}+y{\mathrm{Li}}^{+}+(x+y)e^{-}↔{\mathrm{MnOOH}}_{x}{\mathrm{Li}}_{y}.$$

**Figure 3:**
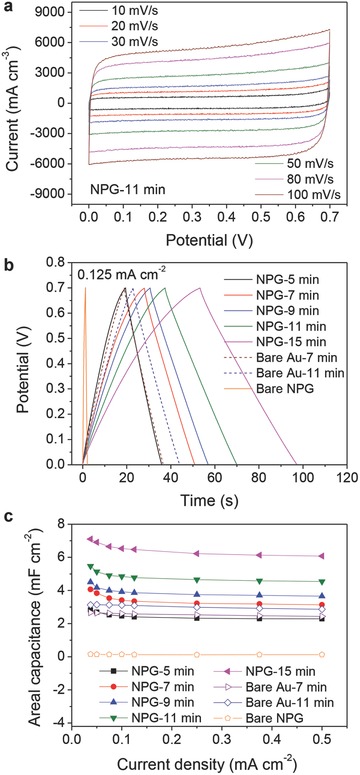
Electrochemical performance of the hierarchical NPG/MnO_2_ MPCs in aqueous liquid electrolyte. a) Cyclic voltammogram profiles obtained at different scan rates on the MPCs (MnO_2_ plating time, 11 min) in 5.0 m LiCl liquid electrolyte. The curves retain a rectangular shape over 0.7 V potential window. b) Galvanostatic charge/discharge curves of the MPCs with various MnO_2_ plating time, all operated at a current density of 0.125 mA cm^−2^ in 5.0 m LiCl. Data for bare hierarchical NPG, bare Au/MnO_2_ MPCs are shown for comparison. c) Areal capacitance of the MPCs as calculated from the charge/discharge curves at different current densities.

The absence of redox peaks indicates that the supercapacitors are charged and discharged at a pseudoconstant rate over the entire voltammetric cycles. Even though the NPG network may also contribute some double‐layer capacitance, the contribution is very small, especially for samples with a high MnO_2_ loading amount (≈5.5% of the overall capacitance for 5 min plating, and ≈2% for 15 min plating). Therefore, the hierarchical NPG mainly acts as the current collector and support of the active MnO_2_.

The MPCs were also tested by galvanostatic charging/discharging (Figure 3b and Figure S10, Supporting Information). Regardless of MnO_2_ loading amounts, the MPCs show near triangular charge/discharge profiles, in accordance with the CV curves as in Figure 3a. The areal capacitance of the MPCs dramatically increases with the plating time from 5 to 15 min (Figure 3c). More importantly, a linear relationship can be found between the areal capacitance and the MnO_2_ plating time/amounts (Figure S11, Supporting Information), indicating insignificant capacity trade‐off with increasing MnO_2_ loading amounts. The specific capacitance, normalized to the mass of MnO_2_, shows a small divergence of no more than 50 F g^−1^_MnO2_ (Figure S11, Supporting Information), suggesting an efficient utilization of the active material. For comparison, MSCs constructed by MnO_2_ directly grown on bare Au templates (bare Au/MnO_2_ MPCs, Figure S12, Supporting Information) were also tested. With the same plating time, the areal capacitance of bare Au/MnO_2_ MPCs is much lower than that of the hierarchical NPG/MnO_2_ MPCs (Figure 3c). The gravimetric capacitance of MnO_2_ in the bare Au/MnO_2_ MPCs is also lower, and decreases significantly when the MnO_2_ plating time increases from 5 to 11 min (Figure S11, Supporting Information). An areal capacitance up to 7.1 mF cm^−2^ can be obtained from the hierarchical NPG/MnO_2_ MPCs, which is much higher than those of electrochemical double‐layer microcapacitors (0.4–2 mF cm^−2^),^2^ carbon/graphene based MSCs (1.7 mF cm^−2^ for onion‐like carbon based MSCs,^2^ 0.51 mF cm^−2^ for laser‐written reduced graphene oxide MSCs^8^) and is also superior to other MnO_2_ based MPCs with a similar electrode thickness (0.2–1.2 mF cm^−2^)^37^, ^38^ (Table S2, Supporting Information).

The hierarchical NPG/MnO_2_ MPCs were also fabricated as all‐solid‐state microdevices using a hydrogel‐polymer PVA‐LiCl electrolyte. The CVs and galvanostatic charging/discharging profiles of the all‐solid‐state microdevices are similar to those of the MPCs with the liquid electrolyte (**Figure** **4**a and Figures S13 and S14, Supporting Information). In the all solid configuration, the specific capacitance of MnO_2_ is ≈10–30 F g^−1^_MnO2_ higher than that in liquid electrolyte MPCs at the same current density. This is probably due to a higher salt concentration in the electrolyte as the result of gel drying. A stack capacitance up to 99.1 F cm^−3^, corresponding to an energy density of 6.74 mW h cm^−3^, has been obtained from the solid‐state hierarchical NPG/MnO_2_ MPCs with a MnO_2_ plating time of 11 min (Figure 4b), which is much higher than the literature values of the best carbon/graphene‐based MSCs (1.3 F cm^−3^ for onion‐like carbon based MSCs,^2^ 3.1 F cm^−3^ for laser‐written reduced graphene oxide MSCs,^8^ 3.05 F cm^−3^ for laser‐scribed graphene MSCs,^9^ 17.9 F cm^−3^ for reduced graphene MSCs^10^ and 6.1 F cm^−3^ for reduced graphene oxide/carbon nanotube MSCs^12^) and pseudocapacitive microdevices (16.4–39.3 F cm^−3^ for MnO*_x_*/Au multilayer MSCs,^19^ 26.5–66.0 F cm^−3^ for polyaniline nanowire array MSCs,^17^ 8–25 F cm^−3^ for CoO/carbon nanotube MSCs,^39^ 4.42 F cm^−3^ for graphene/MnO_2_/Ag nanowire MSCs^40^ and 30–50 F cm^−3^ for MWCNT/MnO_*x*_ MSCs^41^) (Table S2, Supporting Information). Even at a high current density of 0.5 mA cm^−2^ (0.5 mA cm^−2^ corresponds to about 18.4 A g^−1^_MnO2_ for 7 min plating, and 13.2 A g^−1^_MnO2_ for 11 min plating), the stack capacitance is as high as 61.5 F cm^−3^ (Figure 4b). The high power performance of the MPCs was further confirmed by electrochemical impedance spectroscopy (EIS) (Figure 4c,d). The MPC shows a pure capacitive behavior at frequencies lower than 3 Hz and is characterized by a small relaxation time constant τ_0_ of 200 ms which is much lower than that of other MnO_2_ based supercapacitors (500 ms for carbon nanoparticles/MnO_2_ nanorods hybrid structure,^42^ 3.2–8.0 s for MnO_2_/carbon composites^43^). The high rate capability and excellent frequency response of the MPCs originate from the highly accessible surface of MnO_2_ from the open nanoporous structure of the hybrid electrodes. The hierarchical NPG/MnO_2_ MPCs are also very stable, showing 90% capacitance retention over 1000 repeated cycles and 87% over 1500 cycles (Figure S15, Supporting Information).

**Figure 4:**
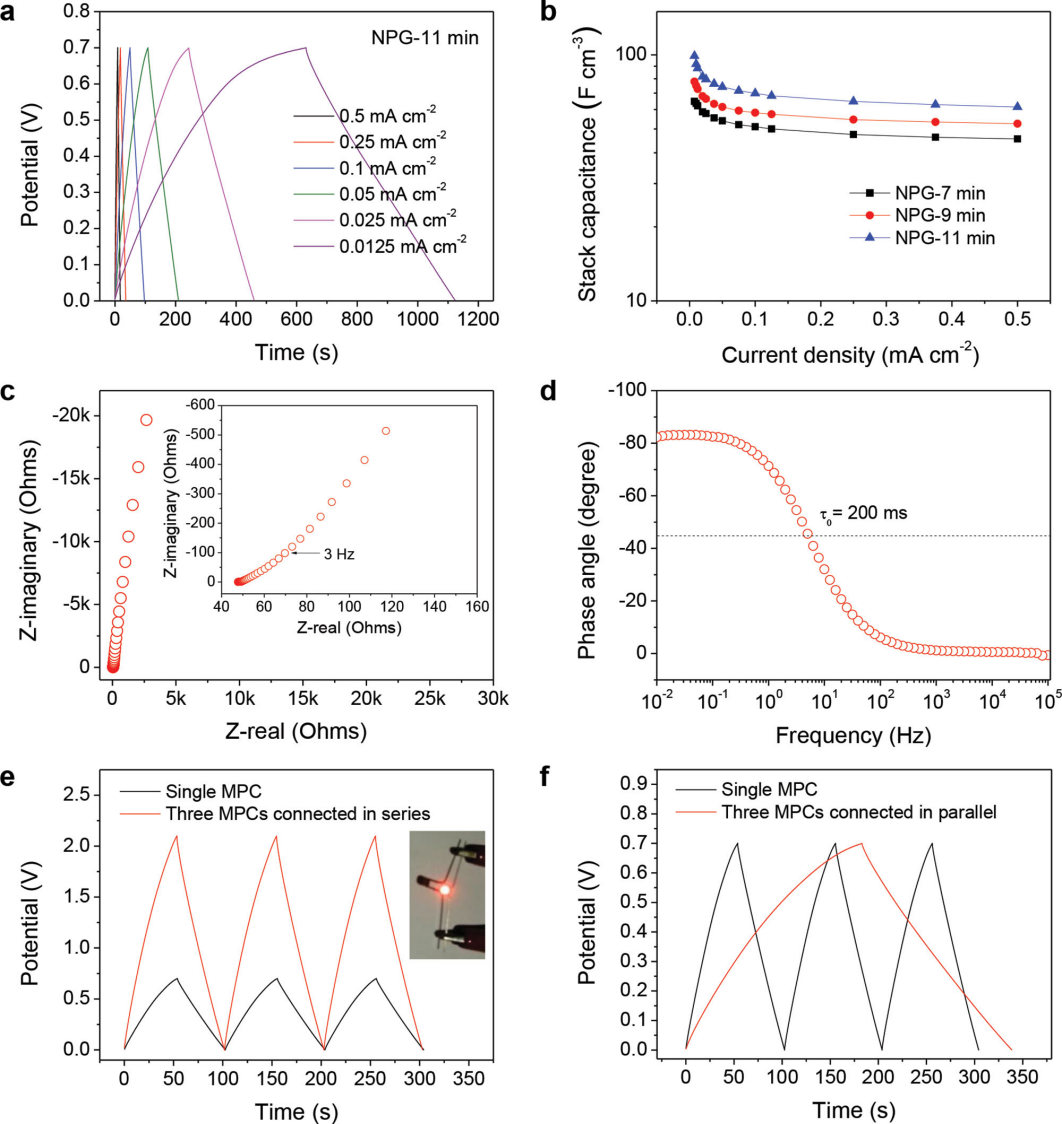
Electrochemical performance of the hierarchical NPG/MnO_2_ MPCs in aqueous solid‐state electrolyte. a) Galvanostatic charge/discharge curves of the solid‐state MPCs with a PVA‐LiCl electrolyte at various current densities. MnO_2_ plating time is 11 min. b) Volumetric stack capacitance of the solid‐state MPCs with various MnO_2_ plating time as a function of the current density. c) Complex plane plot of the impedance of a solid‐state MPCs. Inset is a magnified plot of the high‐frequency region. d) Impedance phase angle as a function of frequency for the solid‐state MPCs. e,f) Galvanostatic charge/discharge curves for three solid‐state MPCs (MnO_2_ plating time, 11 min) connected in series (e) and in parallel (f). A single device is shown for comparison. All devices were operated at 0.1 mA cm^−2^. Inset in (e) is a demonstration showing that three MPCs connected in series can be used to power a light‐emitting diode (LED).

Because of the microscale volume, a single device can only provide a limited power, which cannot meet the demand of most practical applications. Therefore, the microdevices need to be assembled together in series or/and parallel combinations to come up with a specific voltage and capacitance rating. Figure 4e,f demonstrates the feasibility of connecting the hierarchical NPG/MnO_2_ MPCs in serial and parallel configurations (see also Figure S16, Supporting Information). By connecting three solid‐state MPCs in series, the output voltage can be amplified by three times while the charging/discharging time is identical to that of a single device (Figure 4e), verifying a very good control over the operating voltage window. As the voltage meets that of a red light‐emitting diode (LED), the LED can be powered by such a MPC combination (Figure 4e, inset). On the other hand, by connecting three MPCs in parallel, the powering life of the MPCs can be extended for three times (Figure 4f).

The hierarchical NPG/MnO_2_ MPCs with either liquid‐ or solid‐state electrolytes described above are all based upon aqueous electrolytes which have an arrow operating voltage window, barely exceeds 1.0 V. As the energy density is proportional to the square of the potential window according to *E* = 1/2 CV^2^, enlarging the potential window will quadratically increase the energy density.^14^, ^44^ Organic electrolytes and ionic liquids have been used in supercapacitors due to their large stability voltage window. In this study, an ionogel, prepared by mixing 1‐ethyl‐3‐methylimidazolium dicyanamide (EMI‐DCA) ionic liquid (Table S3, Supporting Information) with fumed silica nano‐powders, was used for preparing the nonaqueous solid‐state NPG/MnO_2_ MPCs. Electrochemical performance of the ionogel‐based solid‐state MPCs is shown in **Figure** **5**. It can be seen that the change from aqueous to ionic liquid electrolyte increases the operable cell voltage from 0.7 to 2.1 V. Although the microdevices exhibit resistive behavior, the CVs retain a rectangular shape over scan rates from 10 to 800 mV s^−1^, indicating a good high‐rate performance (Figure 5a). The galvanostatic discharge curves of the microdevices with EMI‐DCA ionogels show a relatively quick voltage drop at the initial stage of the discharge process (Figure 5b, inset), which is mainly due to the large resistance of the ionic liquid. Stack capacitance of the microdevice was calculated according to the formula^45^

(1) $$C_{v}=\frac{C_{MPC}}{V}=\frac{2i\int Edt}{V{\left(\Delta E\right)}^{2}}$$

where *i* is the applied current (in A), *E* is the potential (in V), Δ*E* is the operating voltage window (in V) and *V* is the stack volume of the microdevice (in cm^3^). A capacitance of 20.8 F cm^−3^ can be obtained from the ionogel‐based MPCs at 0.25 mA cm^−2^ (Figure 5b), which is only 1/3 of that for PVA‐LiCl based microdevices (64.7 F cm^−3^) at the same current density (Table S4, Supporting Information). The capacitance degradation in ionic liquids, as compared with in aqueous electrolytes, is a common issue, and mainly originates from the low ionic conductivity of the ionic liquid electrolytes (only a few milliSiemens per centimeter at room temperature, Table S2, Supporting Information).^14^ However, the stack capacitance of the hierarchical NPG/MnO_2_ MPCs is still one order of magnitude higher than that of the reported graphene‐based MSCs, 1.40–2.35 F cm^−3^, with an ionogel electrolyte.^9^ In addition, because of the threefold increase in the operating voltage, the ionogel‐based MPCs possess a high energy density of 12.7 mW h cm^−3^ at 0.25 mA cm^−2^, which is about three times higher than that of PVA‐LiCl based MPCs (4.4 mW h cm^−3^ at 0.25 mA cm^−2^). Moreover, the high capacitances of 16.0 F cm^−3^ and 9.1 F cm^−3^ can be maintained at 1.25 mA cm^−2^ and 10.0 mA cm^−2^, respectively (Figure 5b), indicating that the MPCs can work well at ultrahigh current densities.

**Figure 5:**
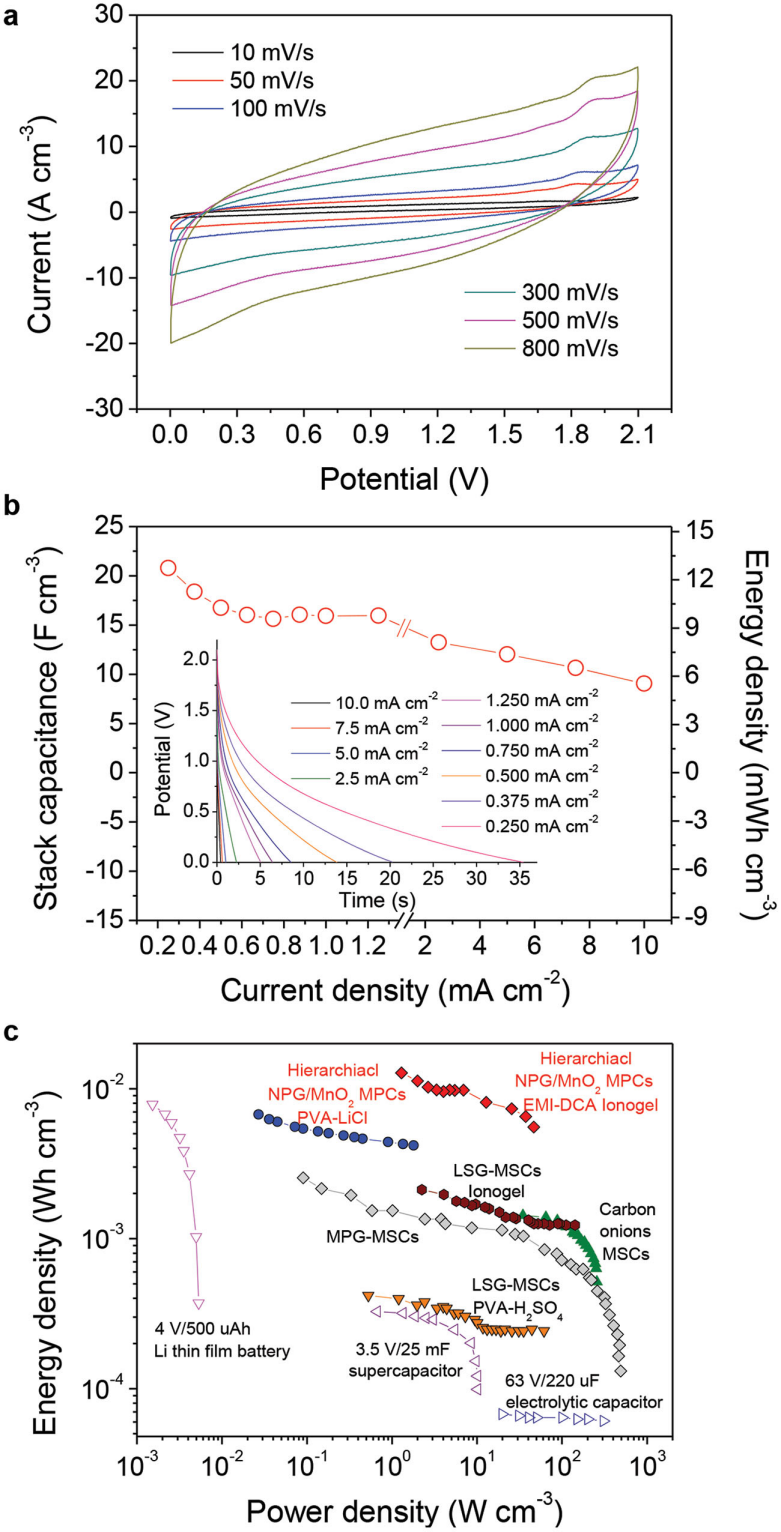
Electrochemical performance of the solid‐state hierarchical NPG/MnO_2_ MPCs in EMI‐DCA ionogel electrolyte. a) Cyclic voltammogram profiles of the MPCs at various scan rates between 10 and 800 mV s^−1^. b) Volumetric stack capacitance and energy density of the MPCs as a function of the charge/discharge current density. Inset shows the galvanostatic discharge curves of the MPCs at various current densities. c) Ragone plot (energy density versus power density) of the hierarchical NPG/MnO_2_ MPCs. Other MSCs reported in the literature^2^, ^9^, ^10^ and commercially available energy‐storage systems are shown for comparison.

Figure 5c shows the Ragone plot in which the stack energy and power densities of the hierarchical NPG/MnO_2_ MPCs are compared with those of commercially available energy‐storage systems and other best MSCs reported in the literature. Remarkably, the hierarchical NPG/MnO_2_ MPCs show a comparable power density as the commercial electrolytic capacitors and conventional carbon based supercapacitors, but can deliver two orders of magnitude higher energy density. The high energy density is even better than the commercial Li thin film batteries while the hierarchical NPG/MnO_2_ MPCs have the power density three orders of magnitude higher than that of the Li thin film batteries. The stack capacitance of the hierarchical NPG/MnO_2_ MPCs is about two orders of magnitude higher than carbon/graphene‐based MSCs, giving rise to more than one order of magnitude higher specific energy (up to 12.7 mW h cm^−3^) with a high power density (up to 46.6 W cm^−3^) comparable to that of the carbon/graphene‐based MSCs.

The outstanding supercapacitor performance for the hierarchical NPG/MnO_2_ MPCs originates from the full utilization of the pseudocapacitive MnO_2_ in an interdigital 3D configuration, which highlights the key merits of the hierarchical nanoporous hybrid electrodes. First, the large porosity of hierarchical NPG together with an efficient mass transport can accommodate a large amount of active materials within the NPG framework for high specific and areal capacitance. Second, the bicontinuous and highly conductive porous structure with interconnected open pores allows an easy access of the ions, and thus facilitates the efficient utilization of the active material and a high power delivery. Third, the high surface area of the hierarchical NPG helps maximize the area of contact junctions for both electrolytes and active materials.

All the techniques involved in the fabrication of hierarchical NPG/MnO_2_ MPCs, including the electrochemical plating of precursor alloys, dealloying and microfabrication for patterning, are adopted from existing MEMS techniques. Hence, the MPCs can be directly integrated into the electric circuits of MEMS as on‐chip power supply without additional assembly and integration and is ready to scale up to industry level for mass production at a lower cost in processing. Together with unprecedented capacitive properties of concurrently realized high energy and power delivery, the hierarchical NPG/MnO_2_ MPCs are very promising candidates as on‐chip micro power supply for miniaturized MEMS and electronic devices.

In summary, we have successfully developed an on‐chip micro‐pseudocapacitor based on hierarchical nanoporous hybrid electrodes consisting of 3D bicontinuous NPG and pseudocapacitive MnO_2_. The novel structure design offers the hierarchical NPG/MnO_2_ micro‐pseudocapacitors an ultrahigh specific energy of 12.7 mW h cm^−3^, better than the state‐of‐the‐art thin film Li ion batteries, and a high power density of 46.6 W cm^−3^, comparable to carbon/graphene‐based micro‐supercapacitors. Importantly, the hierarchical NPG/MnO_2_ micro‐pseudocapacitors are fabricated by a standard MEMS method and can be directly integrated into the electric circuits of MEMS as on‐chip power supply without additional assembly and integration. This study shines a light on the realization of a high‐performance micro‐supercapapcitor towards practical applications and may have an important implication in developing high performance microdevices by designing hierarchical porous structure.

## Experimental Section

*Fabrication of Hierarchical NPG and Assembly of the MPCs*: The fabrication process of the hierarchical NPG/MnO_2_ MPCs is schematically illustrated in Figure S1, Supporting Information. A thin film of Au/Cr was first sputtered onto a 1‐mm thick soda lime glass slide and then machined, by the microfabrication technique, into an interdigital configuration (gold template) that consists of 16 fingers, every eight out of which were electrically connected. Hierarchical NPG microelectrodes were prepared by multistep dealloying AuAg alloy precursors beforehand deposited on the gold templates. The AuAg alloy was electrochemically plated from a magnetic stirred KAu(CN)_2_/KAg(CN)_2_ (6 × 10^–3^/23.5 × 10^–3^ m, with 0.05 m Na_2_CO_3_) mixture solution by applying a constant potential of −1.15 V versus Ag/AgCl on the gold templates for a duration of 2 min. NPG with hierarchical porosity was then created by subjecting the alloy precursor to a preliminary dealloying which partly removed the Ag from the alloy, followed by coarsening at high temperature and a final extraction of Ag. Electrochemical dealloying at 0.805 V versus Ag/AgCl in 1.0 m HNO_3_ for 900 s and chemical dealloying in concentrated HNO_3_ (69 wt%) for 5 h were used for the first and second step dealloyings, respectively. The coarsening was carried out inside a 300 °C tube furnace with Ar protection (500 sccm) for 1 h. MnO_2_ was plated from a solution containing 0.2 m Mn(CH_3_COO)_2_ 4H_2_O and 0.2 m Na_2_SO_4_ by running a potential sequence of 0.40 V–5 s/0.45 V–10 s versus Ag/AgCl on the NPG electrodes. The hydrogel‐polymer electrolyte, PVA‐LiCl, was prepared by mixing 4 g PVA with 40 mL 5.0 m LiCl aqueous solution. The mixture was heated at ≈90 °C under constant stirring until a clear and transparent gel‐like solution was obtained. The EMI‐DCA ionogel was prepared by mixing fumed silica nano‐powders with the ionic liquid EMI‐DCA at a mass ratio of 0.4:5.7 g, and was left stirring for 5 h in an Ar‐filled glove box to produce a clear viscous solution.

*Characterization and Electrochemical Measurement*: The microstructure and chemical composition of hierarchical NPG, NPG/MnO_2_ and the microdevices were investigated using a field‐emission scanning electron microscope (JEOL JIB‐4600F, 15 keV) equipped with an X‐ray energy‐dispersive spectroscopy (EDS). The electrochemical performance of the MPCs was investigated by cyclic voltammetry, galvanostatic charge/discharge tests and electrochemical impedance spectroscopy using a VersaSTAT3 electrochemical workstation (Princeton Applied Research, USA) at room temperature.

## Supporting information

As a service to our authors and readers, this journal provides supporting information supplied by the authors. Such materials are peer reviewed and may be re‐organized for online delivery, but are not copy‐edited or typeset. Technical support issues arising from supporting information (other than missing files) should be addressed to the authors.

Supplementary
