# Supplementary material for: On‐Chip Micro‐Pseudocapacitors for Ultrahigh Energy and Power Delivery
Source: Adv Sci (Weinh). 2015 Apr 2;2(5):1500067. doi: 10.1002/advs.201500067 (PMC5115376; doi:10.1002/advs.201500067)
Supplement: Supplementary file 1 — Supplementary [file ADVS-2-0f-s001.pdf]

Copyright WILEY-VCH Verlag GmbH & Co. KGaA, 69469 Weinheim, Germany, 2015.

ADVANCED SCIENCE
Open Access

Supporting Information

for *Adv. Sci.*, DOI: 10.1002/advs.201500067

On-Chip Micro-Pseudocapacitors for Ultrahigh Energy and Power Delivery

*Jiuhui Han, Yu-Ching Lin, Luyang Chen, Yao-Chuan Tsai, Yoshikazu Ito, Xianwei Guo, Akihiko Hirata, Takeshi Fujita, Masayoshi Esashi, Thomas Gessner, and Mingwei Chen**

Copyright WILEY-VCH Verlag GmbH & Co. KGaA, 69469 Weinheim, Germany, 2015.

Supporting Information

On-Chip Micro-Pseudocapacitors for Ultrahigh Energy and Power Delivery

*Jiuhui Han, Yu-Ching Lin, Luyang Chen, Yao-Chuan Tsai, Yoshikazu Ito, Xianwei Guo, Akihiko Hirata, Takeshi Fujita, Masayoshi Esashi, Thomas Gessner, and Mingwei Chen**

J. H. Han, Dr. Y. C. Lin, Dr. L. Y. Chen, Dr. Y. C. Tsai, Dr. Y. Ito, Dr. X. W. Guo, Dr. A. Hirata, Dr. T. Fujita, Prof. M. Esashi, Prof. T. Gessner, Prof. M. W. Chen
WPI Advanced Institute for Materials Research, Tohoku University, Sendai 980-8577, Japan.
E-mail: mwchen@wpi-aimr.tohoku.ac.jp
Dr. Y. C. Tsai
MEMSCORE Cooperation, Sendai 981-3206, Japan
Prof. M. Esashi
Micro System Integration Center, Tohoku University, Sendai 980-0845, Japan
Prof. T. Gessner
Fraunhofer Institute for Electronic Nano Systems, Chemnitz 09126, Germany
Prof. M. W. Chen
State Key Laboratory of Metal Matrix Composites, School of Materials Science and Engineering, Shanghai Jiao Tong University, Shanghai 200030, PR China.
Prof. M. W. Chen
CREST, JST, 4-1-8 Honcho Kawaguchi, Saitama 332-0012, Japan

Calculations of the capacitance

All the supercapacitor performance was tested in a symmetrical two electrode configuration. The capacitance of each micro-device was calculated from the galvanostatic charge/discharge curves at different current densities using the formula:

$$C_{MPC} = \frac{i}{-dE/dt} \quad (1)$$

where *i* is the current applied (in A), *E* is the potential (in V), and *-dE/dt* is the slope of the discharge curve (in V s^{-1}).

Specific capacitance was calculated based on the area or volume of the device stack according to the following formulas:

Areal capacitance:

$$C_A = \frac{C_{MPC}}{A} \quad (2)$$

Volumetric stack capacitance:

$$C_V = \frac{C_{MPC}}{V} \quad (3)$$

where A is the entire projected surface area (in cm^2) of the micro-device, which includes the area of the micro-electrodes and the gaps between them; V is the volume (in cm^3) of the micro-device, which can be calculated by multiplying the entire projected surface area of the device (A, 21.5275 mm^2) into the thickness of the micro-electrodes.

The gravimetric capacitance normalized by the mass of MnO_2 plated on one electrode (C_{MnO_2}) was calculated according to the formula:

$$C_{MnO_2} = \frac{4C_{MPC}}{M} \quad (4)$$

where M is the total mass of MnO_2 loaded on both electrodes.

The energy density (E_V, in Wh cm^{-3}) and power density (P_V, in W cm^{-3}) of the micro-device were calculated as follows:

$$E_V = \frac{C_V \times (\Delta E)^2}{2 \times 3600} \quad (5)$$

$$P_V = \frac{E_V \times 3600}{\Delta t} \quad (6)$$

where C_V is the volumetric stack capacitance obtained from Equation (3) (in F cm^{-3}), ΔE is the operating voltage window (in V), and Δt is the discharge time of the micro-device (in second).

The specific capacitance was also calculated from cyclic voltammetry by integrating the discharge current (i) vs. potential (E) plots using the following formula:

$$C_v = \frac{\int i \, dE}{vV\Delta E} \quad (7)$$

where v is the scan rate (in V/s).

Note that for MPCs using ionogel EMI-DCA as the electrolyte, Equation (1) cannot be readily applied because the slope for the galvanostatic discharge curves does not remain constant over the entire range of applied voltage. Thus, for MPCs based on ionogel EMI-DCA, different formulas were used for calculating specific capacitance and energy density[1]:

$$C_v = \frac{C_{MPC}}{V} = \frac{2i\int Edt}{V(\Delta E)^2} \quad (8)$$

$$E_v = \frac{i\int Edt}{3600V} \quad (9)$$

The current densities for galvanostatic charge/discharge were calculated based on the total surface of the positive and negative micro-electrodes (not including the interspaces between them).

Capacitance values taken from references were also calculated in volumetric stack capacitance (which includes the volumes of both the micro-electrodes and the gaps). In case that the originally reported capacitance does not count the volume of the gaps, the capacitance was recalculated basing on the parameter provided in their texts.

Table S1. Dimensions of the hierarchical NPG/MnO_2 MPCs.

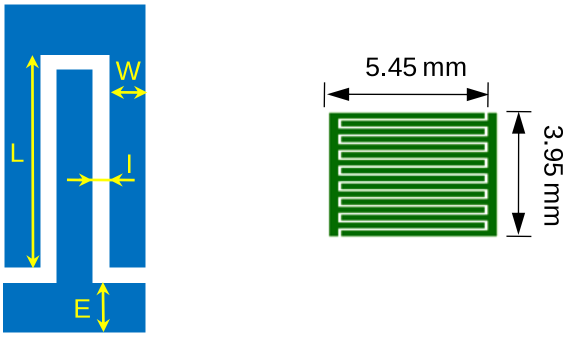

Number of interdigital electrodes	16
Width, W	200 μm
Length, L	4800 μm
Interspace, I	50 μm
Edge, E	300 μm
Total surface area (Include gaps, exclude contact pads)	3.95 mm × 5.45 mm = 21.5275 mm^2

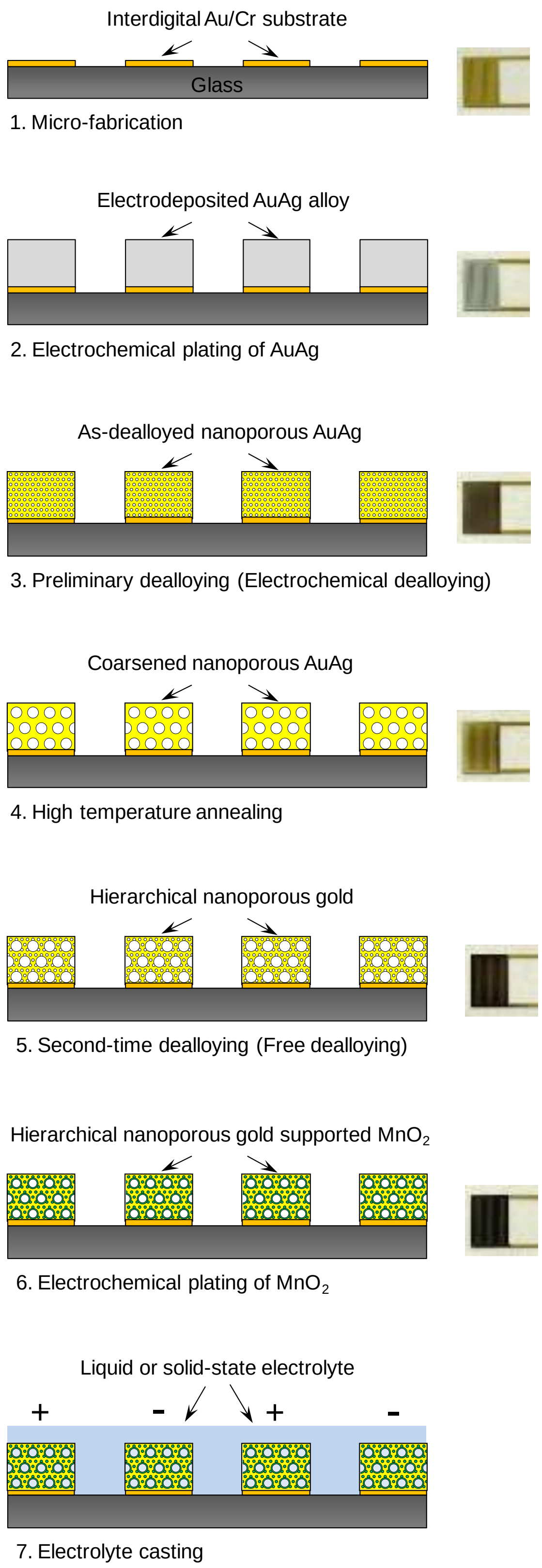

Figure S1. Fabrication procedure of the hierarchical NPG/MnO_2 MPCs. **Step 1:** Interdigital Au/Cr templates on glass substrates were firstly fabricated by a conventional photolithography/etching process. **Step 2:** AuAg alloy precursor films were then grown on these conductive templates by electrochemical plating. **Step 3:** Multi-step dealloying was

used to prepare hierarchical NPG from the AuAg alloy precursor. The first-step dealloying partly removed the Ag by carefully controlling the electrochemical dealloying potential and time, which resulted in a nanoporous AuAg alloy (np-AuAg) with small pores. The residual Ag ratio in the np-AuAg was controlled to be higher than the dealloying parting limit (40-45 at.% Ag) to ensure a feasible second-step dealloying. **Step 4:** The pores and ligaments of the np-AuAg were then coarsened by annealing the np-AuAg at a high temperature. The composition of np-AuAg was also homogenized during this process. **Step 5:** The second-step dealloying in concentrated HNO_3 was conducted to remove almost all the residual Ag and convert the np-AuAg alloy into NPG with a hierarchical porosity. This step can also be replaced by electrochemical dealloying using a high dealloying potential. **Step 6:** MnO_2 was then electrochemically plated into the nano-pores of the hierarchical NPG. **Step 7:** The fabrication process was completed with a final casting of electrolyte, in either a liquid state or a solid state, onto the hierarchical NPG/MnO_2 micro-electrodes. In addition to the cross-sectional schematics, digital photographs of the micro-device in each step are also provided in the figure.

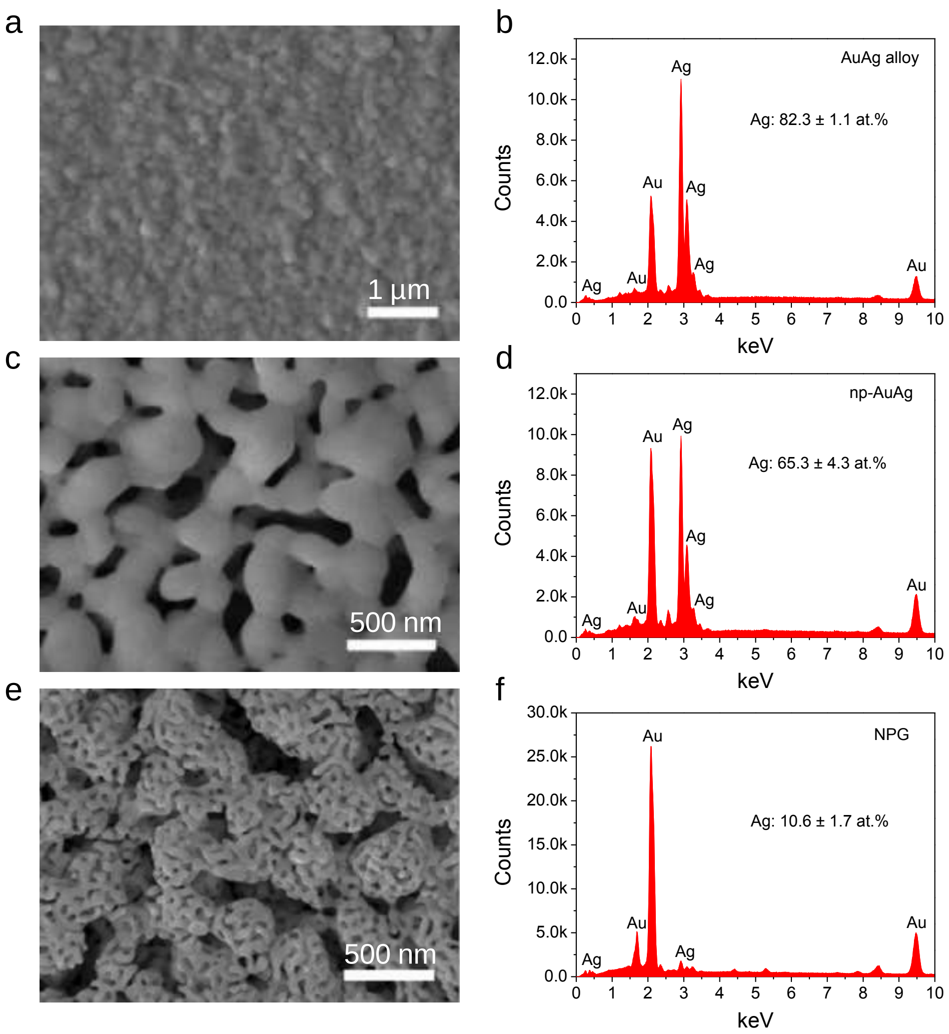

Figure S2. Plane-view SEM images and EDS spectra of AuAg alloy precursor (a,b), coarsened np-AuAg alloy (c,d), and hierarchical NPG (e,f).

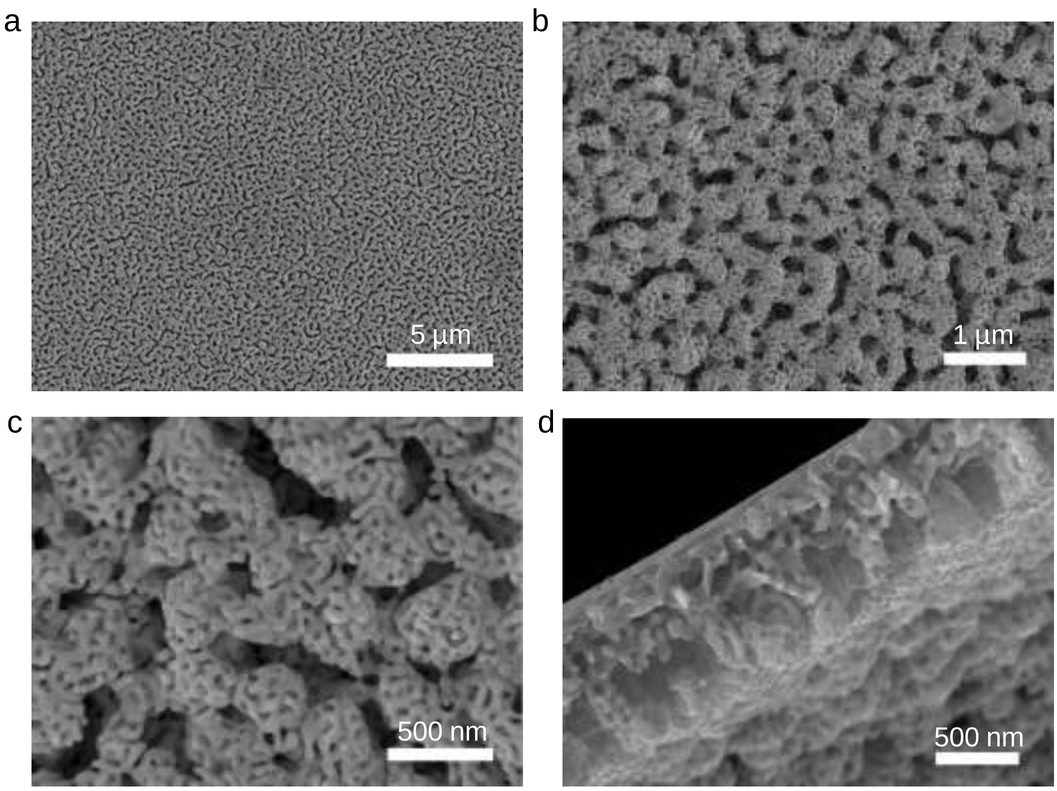

Figure S3. SEM images of hierarchical NPG. a-c, Plane-view, of different magnification. d, Cross-sectional view. The hierarchical NPG is uniform in pore distribution and film thickness, and is also crack-free.

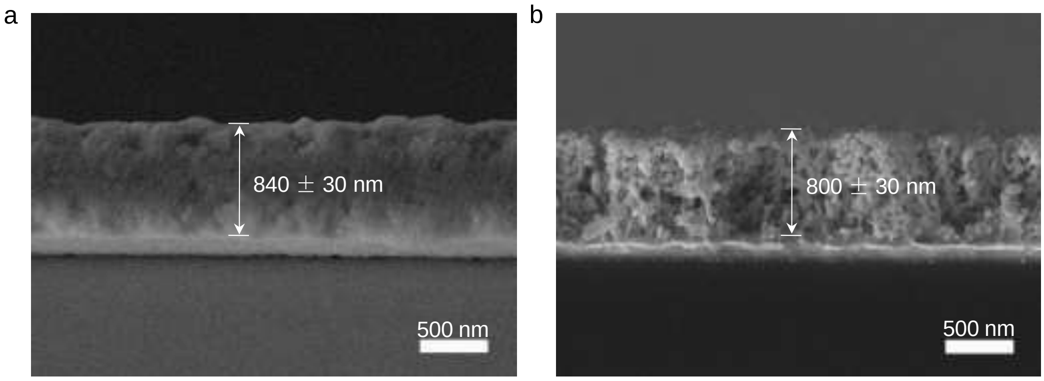

Figure S4. Cross-sectional SEM images of AuAg alloy (a) and hierarchical NPG (b) films. Multi-step dealloying gives rise to very small film thickness shrinkage from the original ~840 nm to the dealloyed ~800 nm.

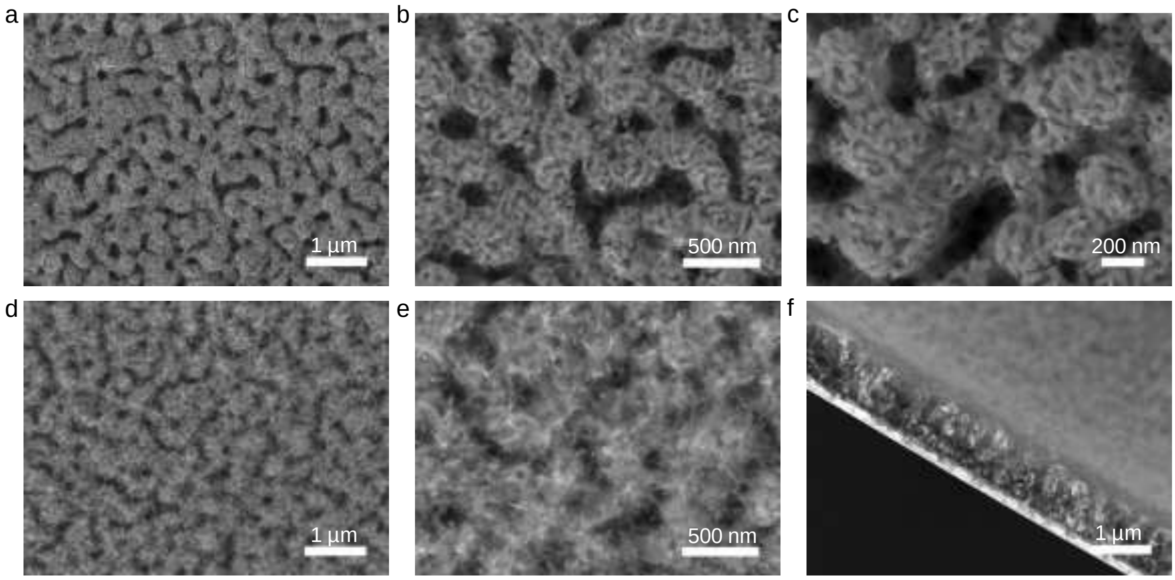

Figure S5. SEM images of hierarchical NPG/MnO_2 composites. a-c, NPG/MnO_2 with a MnO_2 plating time of 11 min. d-f, NPG/MnO_2 with a MnO_2 plating time of 15 min.

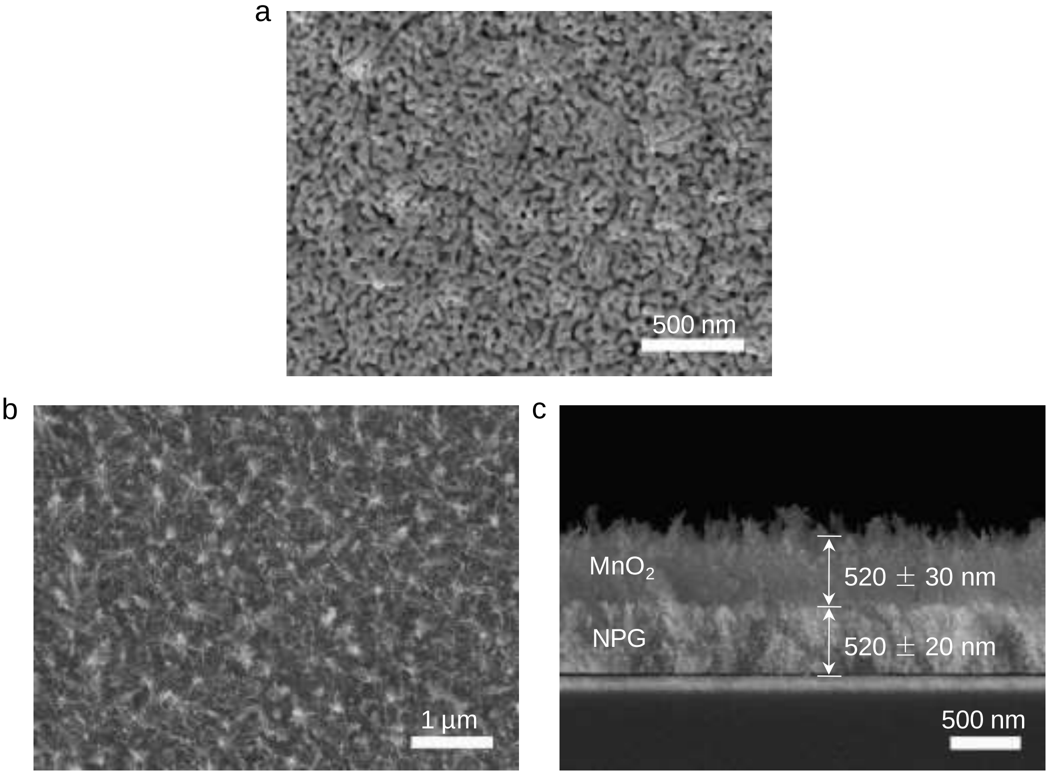

Figure S6. a, Plane-view SEM image of a regular NPG with a monolithic pore size of ~20-30 nm. The NPG was prepared from a $Au_{39.6}Ag_{60.4}$ alloy. b,c, Plane-view and cross-sectional SEM images for MnO_2 plated on the regular NPG film. MnO_2 cannot be uniformly plated into the inner pores of the NPG. A thick MnO_2 film was formed at the NPG top surface. Note that

the overall plating amount (areal density) of MnO_2 is the same as that for 11 min-plated MnO_2 on hierarchical NPG.

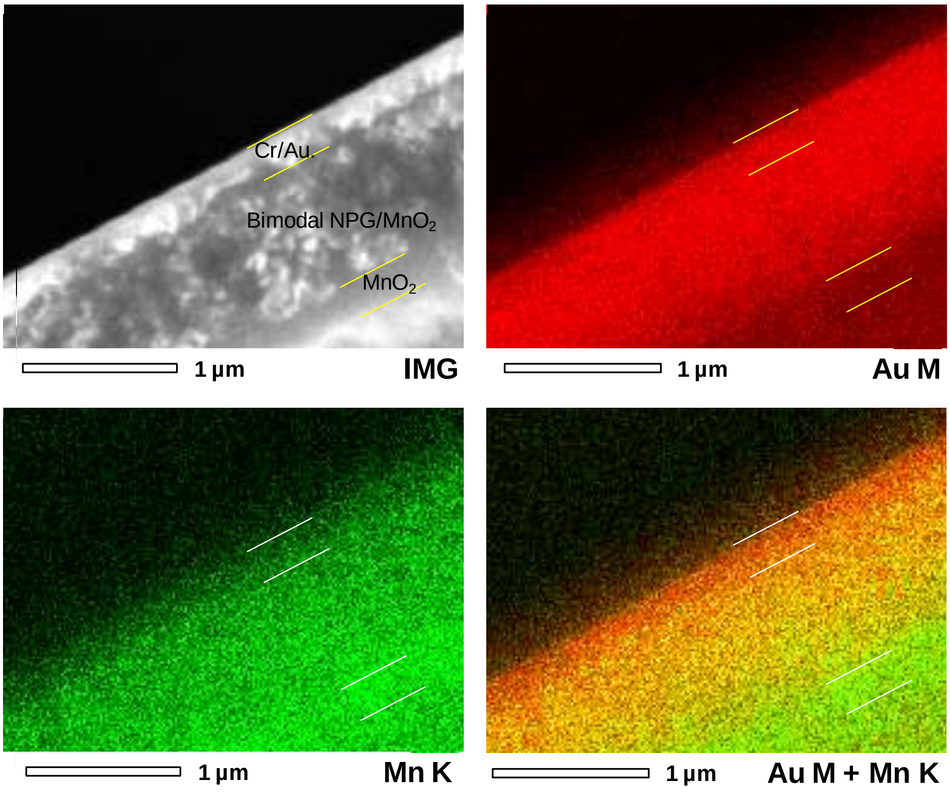

Figure S7. EDS mappings of Au and Mn from the cross-section of hierarchical NPG/MnO_2 composites (plating time 15 min). Mn signals can be detected along the whole cross-section with little intensity gradient, proving that MnO_2 has been successfully plated into the inner pores of the hierarchical NPG films.

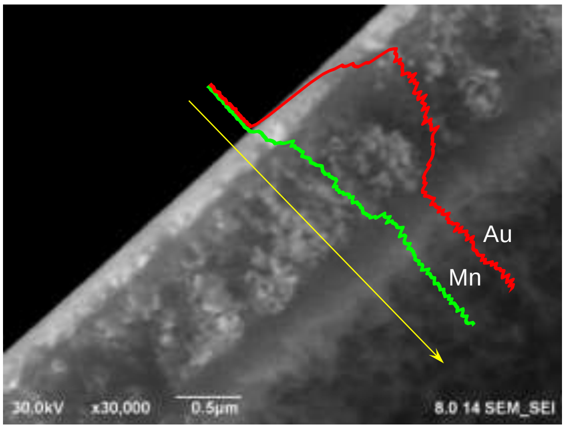

Figure S8. EDS line sweeps of Au and Mn from the cross-section of hierarchical NPG/MnO_2 composites (plating time 15 min).

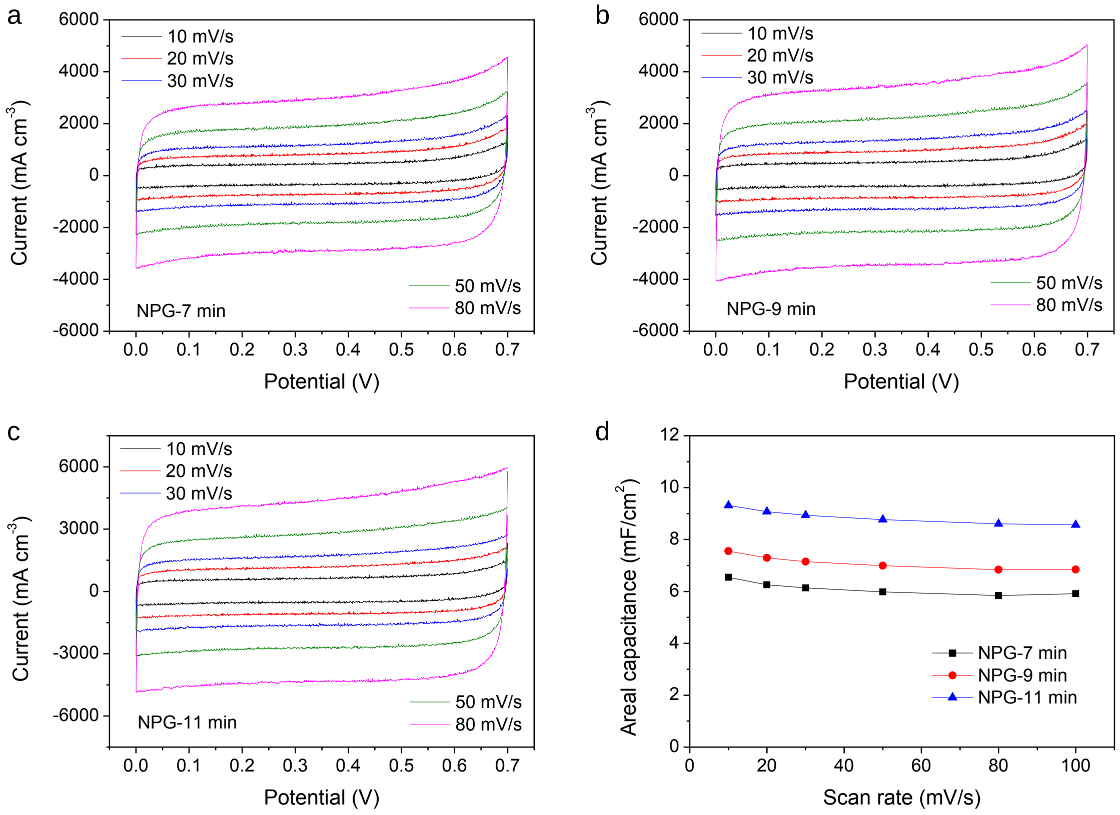

Figure S9. a-c, Cyclic voltammogram profiles of hierarchical NPG/MnO_2 MPCs with different MnO_2 plating time in 5.0 M LiCl liquid electrolyte. d, Areal capacitance calculated from the CV profiles as a function of the scan rate.

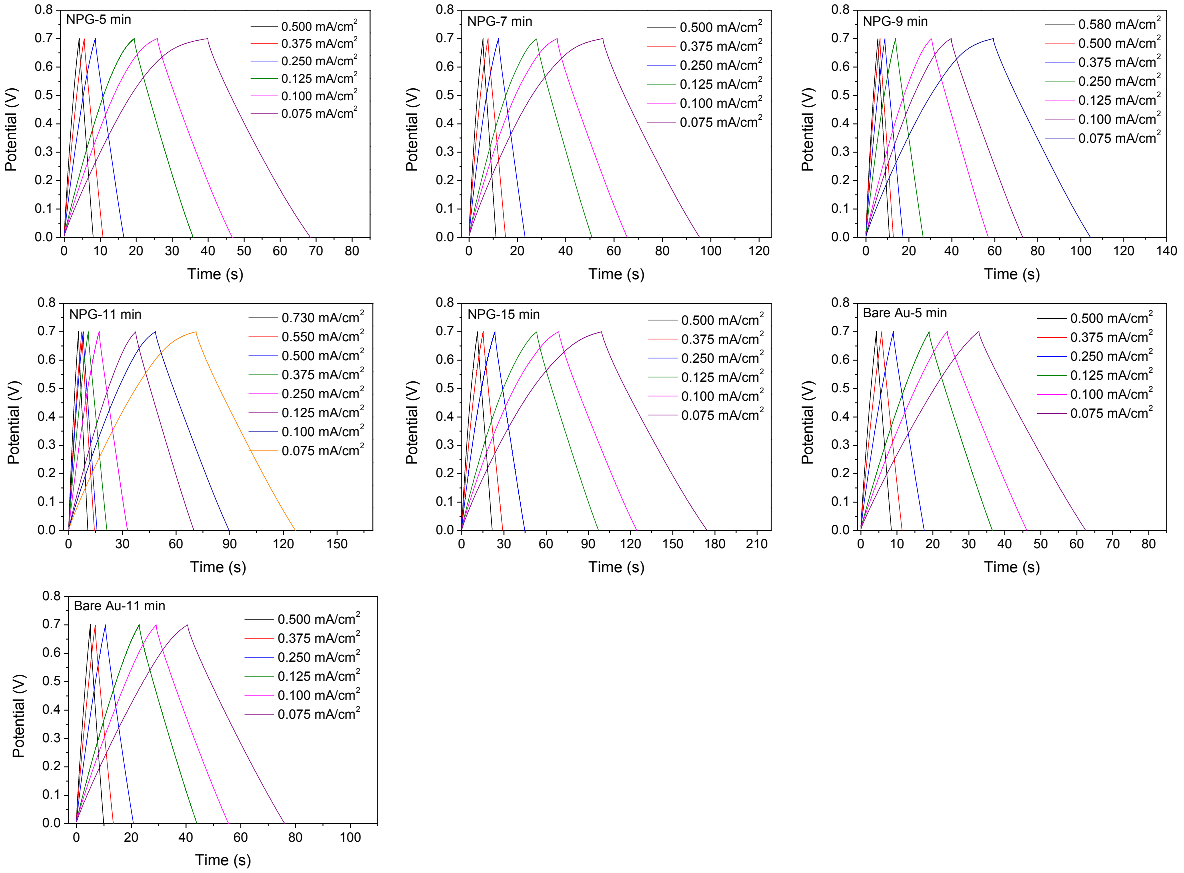

Figure S10. Galvanostatic charge/discharge curves of hierarchical NPG/MnO_2 MPCs and bare Au/MnO_2 MPCs with different MnO_2 plating time in 5.0 M LiCl liquid electrolyte.

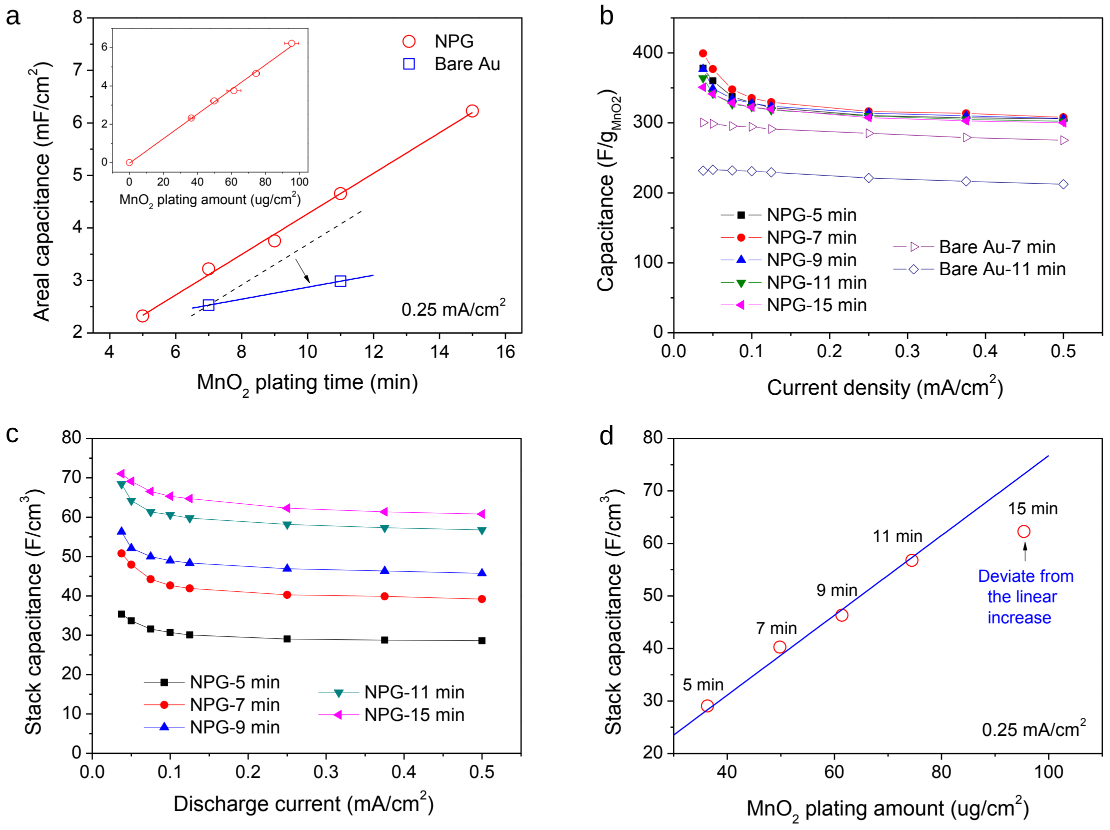

Figure S11. Specific capacitance of the hierarchical NPG/MnO_2 MPCs in 5.0 M LiCl liquid electrolyte. a, Areal capacitance as a function of MnO_2 plating time/amounts. A linear relationship can be found between the areal capacitance and the MnO_2 plating time/amounts for the hierarchical NPG/MnO_2 MPCs, demonstrating small capacity trade-off with increasing loading of the active material. b, Gravimetric capacitance normalized by the mass of MnO_2 as a function of current density. The specific capacitance of the hierarchical NPG/MnO_2 MPCs is ~300-400 F/g_{MnO2}, which is higher than those of the bare Au/MnO_2 MPCs (270-300 F/g_{MnO2} for 7 min plating, and 210-230 F/g_{MnO2} for 11 min plating). Even though the specific capacitance of hierarchical NPG/MnO_2 MPCs decreases slightly with increasing MnO_2 plating amounts (from 5 min to 15 min), the change is much smaller than that for bare Au/MnO_2 MPCs. c, Volumetric stack capacitance with various MnO_2 plating time as a function of the current density. d, Stack capacitance as the function of MnO_2 plating amount. Due to the formation of an extra MnO_2 film (~200 nm in thickness) on the NPG top surface which increases the total volume of the entire device, the stack capacitance of the 15 min plated sample deviates from the linear relationship with the MnO_2 plating amounts.

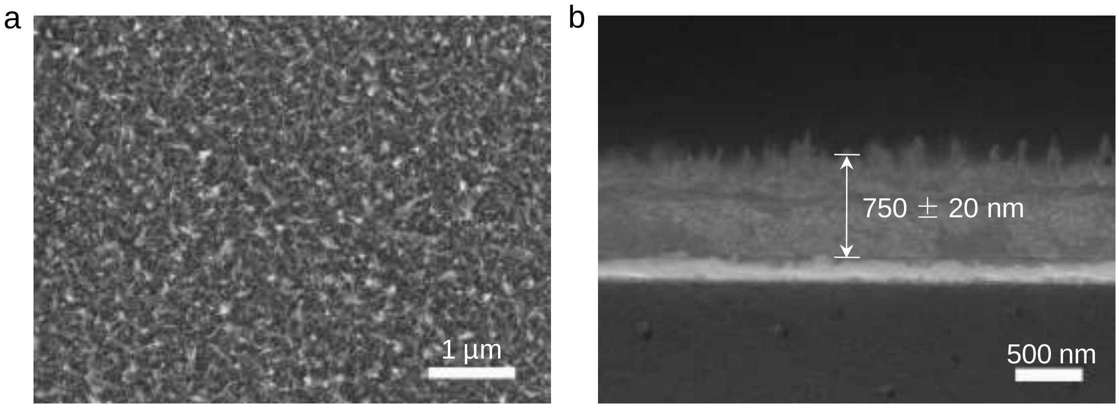

Figure S12. Plane-view and cross-sectional SEM images of a 15 min-plated MnO_2 on bare Au substrate.

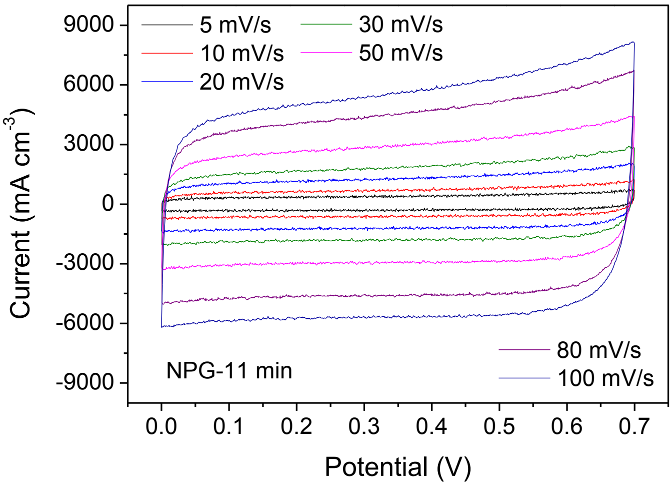

Figure S13. Cyclic voltammogram profiles of a solid-state hierarchical NPG/MnO_2 MPC (MnO_2 plating time, 11 min) with a PVA-LiCl electrolyte at different scan rates.

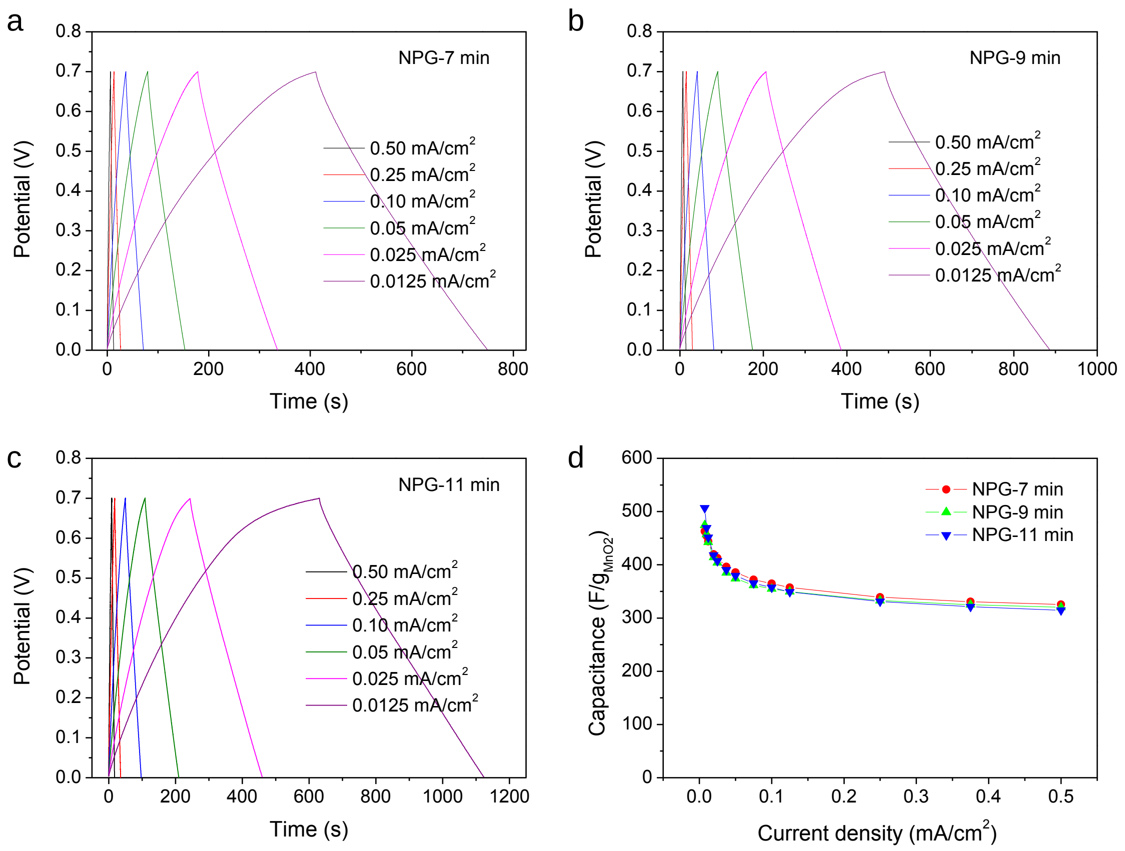

Figure S14. a-c, Galvanostatic charge/discharge curves of hierarchical NPG/MnO_2 MPCs in the solid-state PVA-LiCl electrolyte. d, Gravimetric capacitance as a function of current density.

Table S2. Comparison of hierarchical NPG/MnO_2 hybrid MPCs with the state-of-the-art carbon/graphene based MSCs and other MPCs

Micro-supercapacitors	Electrolyte	Operating voltage (V)	Areal capacitance (mF/cm^2)	Stack capacitance (F/cm^3)	Ref.
Hierarchical NPG/MnO_2 hybrid MPCs	Aqueous LiCl	0.7	7.1	--	This work
Hierarchical NPG/MnO_2 hybrid MPCs	PVA-LiCl	0.7	--	99.1	This work
Onion-like carbon based MSCs	$TEABF_4$/PC	3.0	1.7	1.3	[2]
Laser-written reduced graphene oxide MSCs	Graphene oxide/H_2O	1.0	0.51	3.1	[3]
Laser-scribed graphene MSCs	PVA-H_2SO_4	1.0	2.32	3.05	[4]
Reduced graphene MSCs	PVA-H_2SO_4	1.0	0.08	17.9	[5]
Reduced graphene oxide/carbon nanotube MSCs	Aqueous KCl	1.0	--	6.1	[6]
MnO_x/Au multilayer MSCs	PVA-H_2SO_4	0.8	--	16.4-39.3	[7]
Polyaniline nanowire array MSCs	PVA-H_2SO_4	1.0	--	26.5-66.0	[8]
CoO/carbon nanotube MSCs	PVA-KOH	1.2	--	8-25	[9]
Graphene/MnO_2/Ag nanowire MSCs	Aqueous Na_2SO_4	0.9	--	4.42	[10]
MWCNT/MnO_x MSCs	PVA-H_3PO_4	0.8	--	30-50	[11]

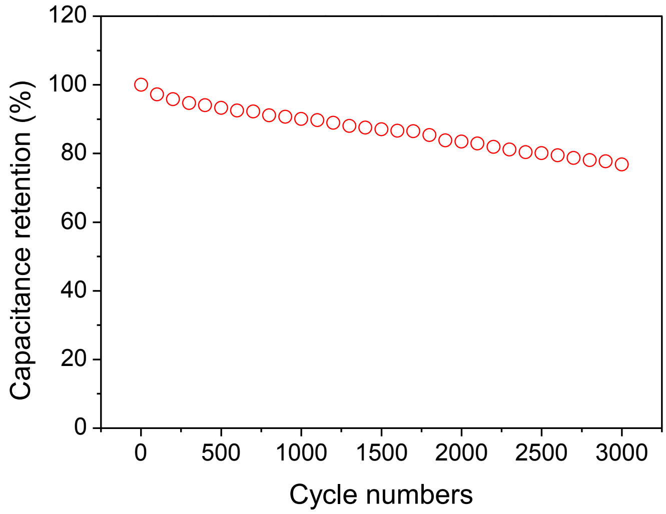

Figure S15. Cycling behavior of the solid-state MPCs (PVA-LiCl electrolyte). The micro-devices were tested in a galvanostatic charge/discharge mode at 0.5 mA/cm^2.

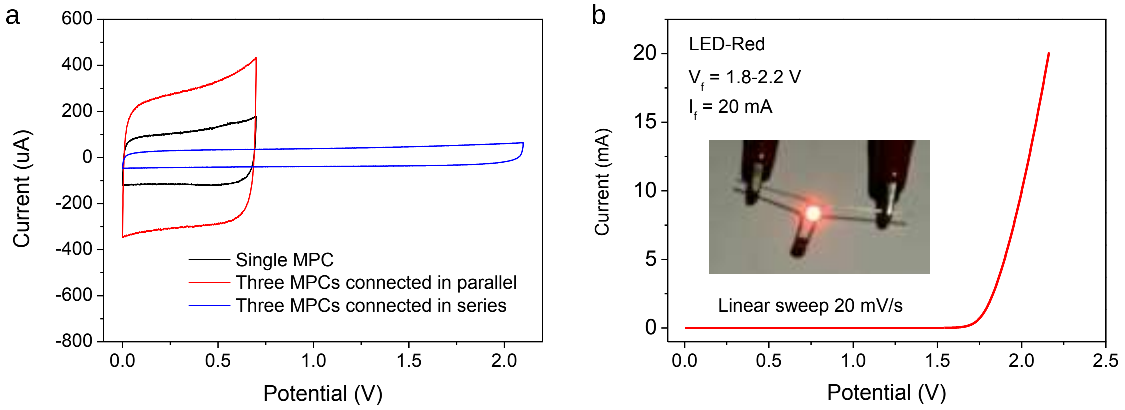

Figure S16. a, Cyclic voltammogram profiles at 100 mV/s for three 11 min-MnO_2-plated solid-state MPCs connected in series and in parallel. A single device is shown for comparison. b, Current-voltage characteristics for the light-emitting diode (LED, red light). Inset shows a digital photograph of the LED powered by three MPCs connected in series.

Table S3. Molecular structure, physical and chemical properties of the 1-ethyl-3-methylimidazolium dicyanamide ionic liquid.

IonLic EMIDCA

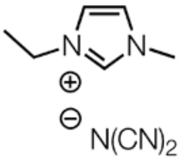

Chemical Name:	1-ethyl-3-methylimidazolium dicyanamide
Molecular Formula:	$C_8H_{11}N_5$
Formula Weight:	177.21 g/mol
Viscosity:	~17 Pa.s (cp) at 22 °C
Electrical conductivity:	~2 E10^{-2} mS/cm
Aspect:	clear transparent liquid

Table S4. Comparison of performance of hierarchical NPG/MnO_2 hybrid MPCs with different electrolytes.

**Electrolyte**	**Operating voltage (V)**	**Current density (mA/cm^2)**	**Stack capacitance (F/cm^3)**	**Energy density (mWh/cm^3)**	**Power dnesity (W/cm^3)**
Aqueous LiCl	0.7	0.0375	68.4	4.7	0.14
		0.25	58.2	4.0	0.90
		0.5	56.8	3.9	1.80
PVA-LiCl	0.7	0.0075	99.1	6.7	0.03
		0.0375	76.3	5.2	0.14
		0.25	64.7	4.4	0.90
		0.5	61.5	4.2	1.80
EMI-DCA ionogel	2.1	0.25	20.8	12.7	1.29
		0.5	16.7	10.3	2.66
		10	9.1	5.6	46.58

All the results are obtained from hierarchical NPG/MnO_2 hybrid MPCs (11 min-MnO_2 plating).

Reference

[1] L.-Q. Mai, A. Minhas-Khan, X. Tian, K. M. Hercule, Y.-L. Zhao, X. Lin, X. Xu, *Nat. Commun.* **2013**, *4*:2923 doi: 10.1038/ncomms3923.

[2] D. Pech, M. Brunet, H. Durou, P. Huang, V. Mochalin, Y. Gogotsi, P.-L. Taberna, P. Simon, *Nat. Nanotechnol.* **2010**, *5*, 651.

[3] W. Gao, N. Singh, L. Song, Z. Liu, A. L. M. Reddy, L. Ci, R. Vajtai, Q. Zhang, B. Wei, P. M. Ajayan, *Nat. Nanotechnol.* **2011**, *6*, 496.

[4] M. F. El-Kady, R. B. Kaner, *Nat. Commun.* **2013**, *4*:1475 doi: 10.1038/ncomms2446.

[5] Z. S. Wu, K. Parvez, X. Feng, K. Müllen, *Nat. Commun.* **2013**, *4*:2487 doi: 10.1038/ncomms3487.

[6] M. Beidaghi, C. Wang, *Adv. Funct. Mater.* **2012**, *22*, 4501.

[7] W. Si, C. Yan, Y. Chen, S. Oswald, L. Han, O. G. Schmidt, *Energy Environ. Sci.* **2013**, *6*, 3218.

[8] K. Wang, W. Zou, B. Quan, A. Yu, H. Wu, P. Jiang, Z. Wei, *Adv. Energy Mater.* **2011**, *1*, 1068.

[9] Y. G. Zhu, Y. Wang, Y. Shi, J. I. Wong, H. Y. Yang, *Nano Energy* **2014**, *3*, 46.

[10] W. Liu, C. Lu, X. Wang, R. Y. Tay, B. K. Tay, *ACS nano* **2015**, DOI: 10.1021/nn5060442.

[11] G. Lee, D. Kim, J. Yun, Y. Ko, J. Cho, J. S. Ha, *Nanoscale* **2014**, *6*, 9655.
